# CRISPR/Cas9-Mediated Deletion of *Foxn1* in NOD/SCID/IL2rg^−/−^ Mice Results in Severe Immunodeficiency

**DOI:** 10.1038/s41598-017-08337-8

**Published:** 2017-08-10

**Authors:** Xinru Wei, Yunxin Lai, Baiheng Li, Le Qin, Youdi Xu, Simiao Lin, Suna Wang, Qiting Wu, Qiubin Liang, Guohua Huang, Qiuhua Deng, Pentao Liu, Donghai Wu, Liangxue Lai, Yao Yao, Peng Li

**Affiliations:** 1 School of Life Sciences, University of Science and Technology of China, Hefei, 230027 China; 2 Key Laboratory of Regenerative Biology, South China Institute for Stem Cell Biology and Regenerative Medicine, Guangzhou Institutes of Biomedicine and Health, Chinese Academy of Sciences, Guangzhou, 510530 China; 3 Guangdong Provincial Key Laboratory of Stem Cell and Regenerative Medicine, South China Institute for Stem Cell Biology and Regenerative Medicine, Guangzhou Institutes of Biomedicine and Health, Chinese Academy of Sciences, Guangzhou, 510530 China; 4 Shenzhen InVivo Biomedicine Co. Ltd, Shenzhen, 518000 China; 5 Department of Respiratory medicine, Nanfang Hospital, Southern Medical University, Guangzhou, 510515 China; 6 Wellcome Trust Sanger Institute, Hinxton, Cambridge, CB10 1HH England UK

## Abstract

Immunodeficient mice engrafted with either normal or cancerous human cells are widely used in basic and translational research. In particular, NOD/SCID/IL2rg^−/−^ mice can support the growth of various types of human cancer cells. However, the hairs of these mice interfere with the observation and imaging of engrafted tissues. Therefore, novel hairless strains exhibiting comparable immunodeficiency would be beneficial. Recently, the CRISPR/Cas9 system has been used for efficient multiplexed genome editing. In the present study, we generated a novel strain of nude NOD/SCID/IL2rg^−/−^ (NSIN) mice by knocking out *Foxn1* from NOD/SCID/IL2rg^−/−^ (NSI) mice using the CRISPR/Cas9 system. The NSIN mice were deficient in B, T, and NK cells and not only showed impaired T cell reconstitution and thymus regeneration after allogeneic bone marrow nucleated cell transplantation but also exhibited improved capacity to graft both leukemic and solid tumor cells compared with NSI, NOG, and NDG mice. Moreover, the NSIN mice facilitated the monitoring and *in vivo* imaging of both leukemia and solid tumors. Therefore, our NSIN mice provide a new platform for xenograft mouse models in basic and translational research.

## Introduction

The development of immunodeficient mice engrafted with human cells or tissues (“humanized” mice) has significantly contributed to translational biomedical research^1–3^. The discovery of athymic nude mice^4^, which was first reported in 1966 as a spontaneously occurring phenotype, enabled the modeling of human tumors in immunodeficient mice^5^. Subsequent improvements include the severe combined immune deficient (SCID)^6^ mutation, targeted mutations in recombination-activating genes 1 and 2 (Rag1^−/−^ and Rag2^−/−^)^7, 8^ that severely cripple the adaptive immune response of the murine host, and a mutation in the gene encoding the common γ chain of the interleukin 2 (IL2) receptor (IL2rg). Mice with a NOD/SCID background with IL2rg mutations, such as NOD.Cg-*Prkdc* ^*scid*^ *Il2rg* ^*tm1Wjl*^ (NSG)^9^ and NODShi.Cg-*Prkdc* ^scid^ *Il2rg* ^tm1Sug^(NOG) mice^10^, are able to grow almost all types of human cancers *in vivo*, including most solid tumors and hematological malignancies, and can be engrafted with functional human immune cells^11–14^. In our previous work, we generated a strain of NOD/SCID/IL2rg^−/−^ (NSI) mice^15–19^. However, the hair present on these strains impaired the observation and imaging of engrafted tumors, necessitating the generation of nude NOD/SCID/IL2rg^−/−^ mice. The nude^4^ gene *Foxn1* (forkhead box N1) encodes a transcription factor for forkhead family proteins^20, 21^. *Foxn1* is continuously expressed in the thymus and is necessary for initial thymus organogenesis and the maintenance of cortical and medullary thymic epithelial cells (cTECs and mTECs)^22^ in both embryonic^23^ and postnatal mice^24–26^. Mutations in *Foxn1* cause inborn thymic dysgenesis and hairless skin^27^.

Various methods have been developed for genome modification, including designer zinc finger nucleases, transcription activator-like effector nucleases, and the type II bacterial CRISPR/Cas9 system. Recently, the CRISPR/Cas9 system has been shown to be suitable for multiplexed genome editing^28, 29^. The ease of design, construction, and delivery of multiple sgRNAs by co-microinjection^30–32^ of Cas9 mRNA suggest that this system can be used to generate a variety of novel immunodeficient mouse strains.

In the present study, we derived a *Foxn1-*mutated NOD/SCID/IL2rg^−/−^ mouse (NSIN) strain using the CRISPR/Cas9 system. Cas9 mRNA and gRNA targeting *Foxn1* were injected into the cytoplasm of pronuclear-stage NSI mouse embryos. The mutant offspring were mated to generate homozygous NSIN mice. The NSIN mice were hairless and deficient in B, T, and NK cells and exhibited an enhanced engrafting capacity for both leukemia and solid tumors compared with NSI, NOG, and NDG mice. Moreover, the hairlessness facilitated tumor observation and imaging. Our study shows that NSIN mice can be used to generate ideal models for basic and translational research.

## Results

### Efficient modification of *Foxn1* in PL08 cells *in vitro* using CRISPR/Cas9

**Figure 1:**
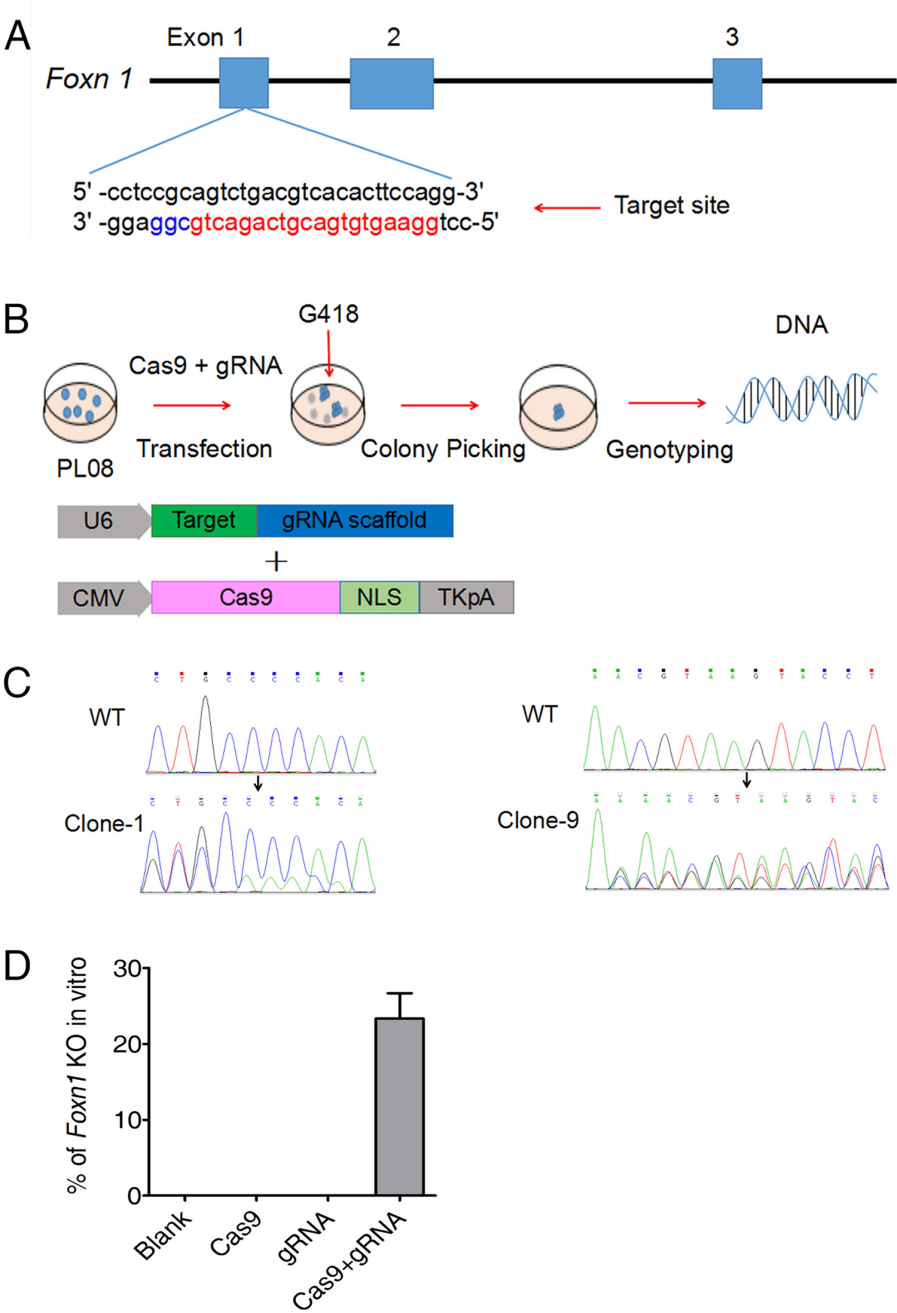
*Foxn1* gene targeting in PL08 cells using a type II CRISPR system *in vitro*. (**A**) Schematic of the Cas9/sgRNA-targeting site in exon 1 of *Foxn1*. The sgRNA-targeting and protospacer-adjacent motif (PAM) sequences are labeled in red and blue, respectively. (**B**) Schematic of *Foxn1 *gene targeting in PL08 cells *in vitro*. PL08 cells were transfected with U6-*Foxn1*-sgRNA vector and pcDNA3.3-hCas9 plasmid. Abbreviations: U6 = U6 polymerase III promoter; CMV = cytomegalovirus promoter; NLS = nuclear localization signal; TK = thymidine kinase; pA = polyadenylation signal. In the plate wells, living cells are shown in blue, and dying cells are shown in gray. (**C**) Genotyping of *Foxn1*-targeted and wild-type (WT) PL08 cells. Clones 1 and 9 are shown. The target exon 1 of *Foxn1* was amplified by PCR and then analyzed using DNA sequencing. Double sequencing peaks in clone 1 showed mutant alleles. The three sequencing peaks in clone 9 may originate from a mixture of two different clones. (**D**) Efficient knock-out of the *Foxn1* gene in PL08 cells by co-transfection with U6-*Foxn1*-sgRNA vectors and pcDNA3.3-hCas9 plasmids. The percentages indicate *Foxn1*-knock-out clones in different groups. The data are representative of three independent experiments. Twenty clones were selected and analyzed in each experiment.

First, to test the targeting accuracy and efficiency of our CRISPR/Cas9 system, we designed gRNA targeting the first exon^33^ of murine *Foxn1* (Fig. 1A) and transfected plasmids expressing mammalian codon-optimized Cas9 and gRNA into a murine PL08 cell line^16^ (Fig. 1B). Twenty-four hours later, transfected cells were selected via a72-h treatment with500 μg/ml G418, and cell clones were then selected. DNA was extracted from twenty cell clones to determine their genotypes in each experiment. DNA sequencing revealed cell clones that carried the expected mutation at the target locus (Fig. 1C). The knock-out efficiency of *Foxn1* in PL08 cells was approximately 20% (Fig. 1D). These data demonstrated the specific and efficient targeting of *Foxn1* by our CRISPR/Cas9 system.

### Generation of NSIN mice by deletion of *Foxn1*

**Figure 2:**
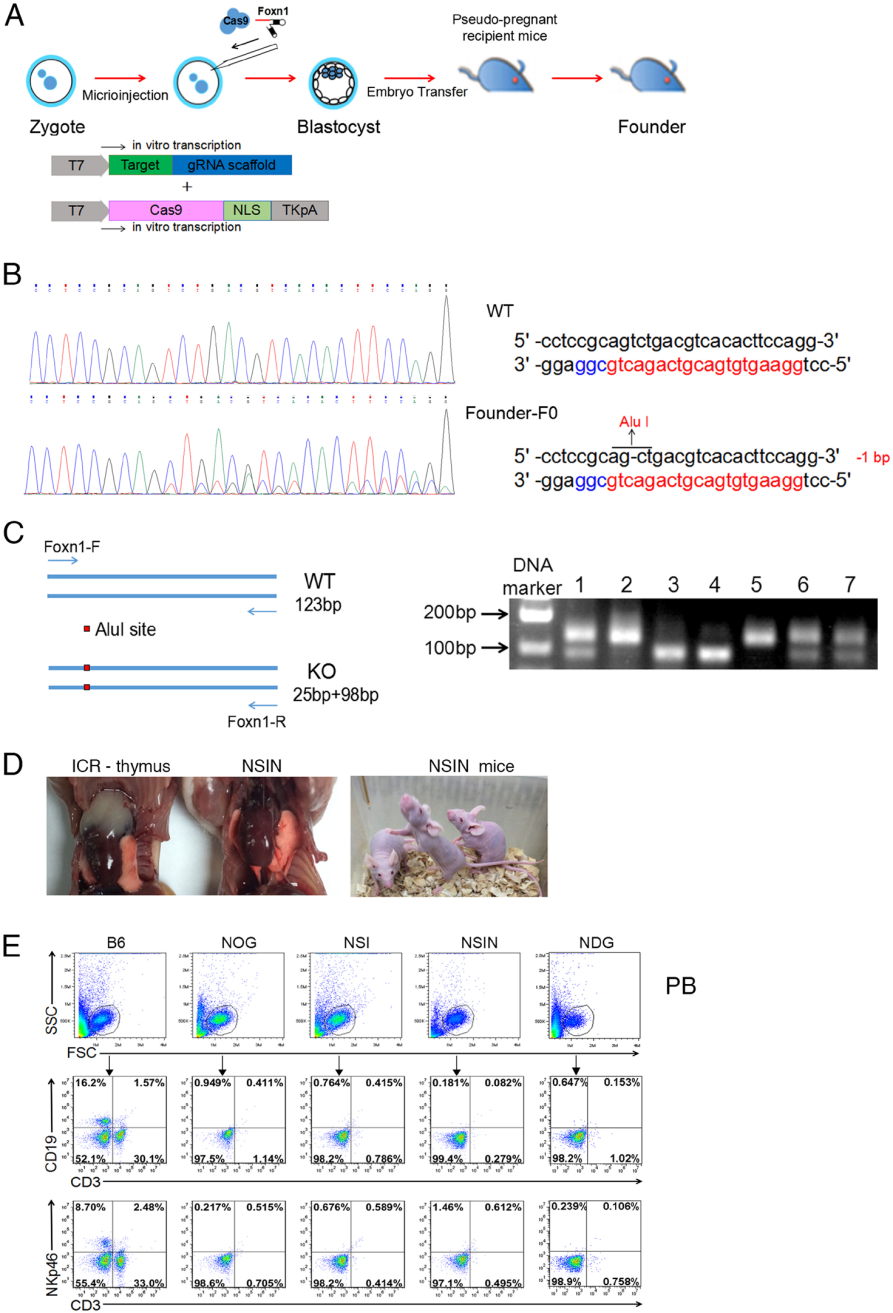
Generation and characterization of Foxn1-deleted NSIN mice. (**A**) Schematic of the generation of NSIN mice. Cas9 mRNA and gRNA were co-injected into the cytoplasm of pronuclear-stage mouse embryos. The injected embryos were then transferred into pseudo-pregnant surrogate mothers. The mouse pups were genotyped and screened for *Foxn1* mutations. The *in vitro* transcription of both vectors was controlled by T7 promoters. (**B**) Sequence analysis of the founder mice. The target exon 1 of *Foxn1* was amplified using PCR and then analyzed by DNA sequencing. The PCR products were 644 bp long. The wild-type (WT) sequence of *Foxn1* is shown at the top of the figure. Double sequencing peaks indicate the presence of mutant alleles in the somatic cells of the mice. The targeting and PAM sequences are labeled in red and blue, respectively. A new AluI enzyme locus was generated by deleting a thymidine residue. (**C**) PCR genotyping of the F2 generation offspring. Primers were designed to amplify 123-bp PCR products spanning the mutation site. The PCR products were digested using the Alu I enzyme. The results from seven offspring mice are shown: WT NSI (lanes 2 and 5), *Foxn1* ^+/−^NSI (lanes 1, 6, and 7), and *Foxn1* ^−/−^NSI (NSIN) mice (lanes 3 and 4). (**D**) Photographs of thymi of ICR and NSIN mice. (**E**) Flow cytometric analysis indicating the absence of B, T, and NK cells in the peripheral blood (PB) of B6, NOG, NSI, NSIN, and NDG mice. Cells were gated from FSC/SSC for the detection of T, B, and NK cells. C57BL/6J (B6) mice were used as a positive control.

Next, using the CRISPR/Cas9 system with multiplexable genome engineering capabilities, we attempted to knock-out the *Foxn1* gene from the NOD/SCID/IL2rg^−/−^ background (NSI mice). *Foxn1-*deleted mice were generated through direct embryo manipulation (Fig. 2A). After *in vitro* transcription, a mixture of Cas9 mRNA (20 ng/μl) and gRNA for *Foxn1* (20 ng/μl) was microinjected into the cytoplasm of pronuclear-stage embryos of NSI mice^28^. Blastocysts derived from the injected embryos were transplanted into foster mothers, and 14 newborn pups were obtained (Table 1). Genomic DNA was extracted from the pups for PCR amplification. DNA sequencing revealed that one mouse carried the expected mutation at the target locus (Fig. 2B). A new AluI restriction enzyme recognition site was generated by deleting a thymidine (Fig. 2B). Then, a 123-bp fragment spanning the target site was amplified using PCR. The PCR products were digested with the AluI enzyme. AluI digestion of the PCR products of wild-type, heterozygous, and homozygous offspring generated fragments with lengths of 123 bp, 123 + 98 + 25 bp, and 98 + 25 bp, respectively (Fig. 2C). Using this restriction fragment length polymorphism (RFLP) assay, we could rapidly and accurately identify the genotypes of offspring mice. Due to the loss of *Foxn1*, homozygous NSIN female mice are poor breeders and fail to lactate. Heterozygous (*Foxn1* ^+/−^NSI) female mice breed well and were mated with homozygous male NSIN mice to generate the NSIN offspring. The average litter size was approximately 6 to 8 pups per litter. Overall, approximately half of the offspring were NSIN mice. The NSIN mice were athymic and hairless (Fig. 2D). Furthermore, B, T, and NK cells were absent in the NSIN mice, similar to NSI, NOG, and NDG mice (Fig. 2E). In summary, we generated a novel strain of nude mice by knocking out the *Foxn1* gene from NOD/SCID/IL2rg^−/−^ (NSI) background mice using CRISPR/Cas9.

**Table 1:** CRISPR/Cas9-mediated *Foxn1* gene targeting in NSI mice.

Gene	Cas9/sgRNA (ng/μl)	Transferred Embryos (Recipients)	Newborn (Dead)	Mutant Alleles per Mouse/Total Mice Tested		
				2	1	0
*Foxn1*	20/20	98(6)	14 (0)	0/14	1/14	13/14

Cas9 mRNA and sgRNA targeting *Foxn1* were injected into fertilized eggs. The blastocysts derived from injected embryos were transplanted into foster mothers and newborn pups were obtained and genotyped. The number of total alleles mutated in each mouse is listed from 0 to 2.

### Loss of *Foxn1* impairs T cell development and thymus regeneration in NSIN mice

**Figure 3:**
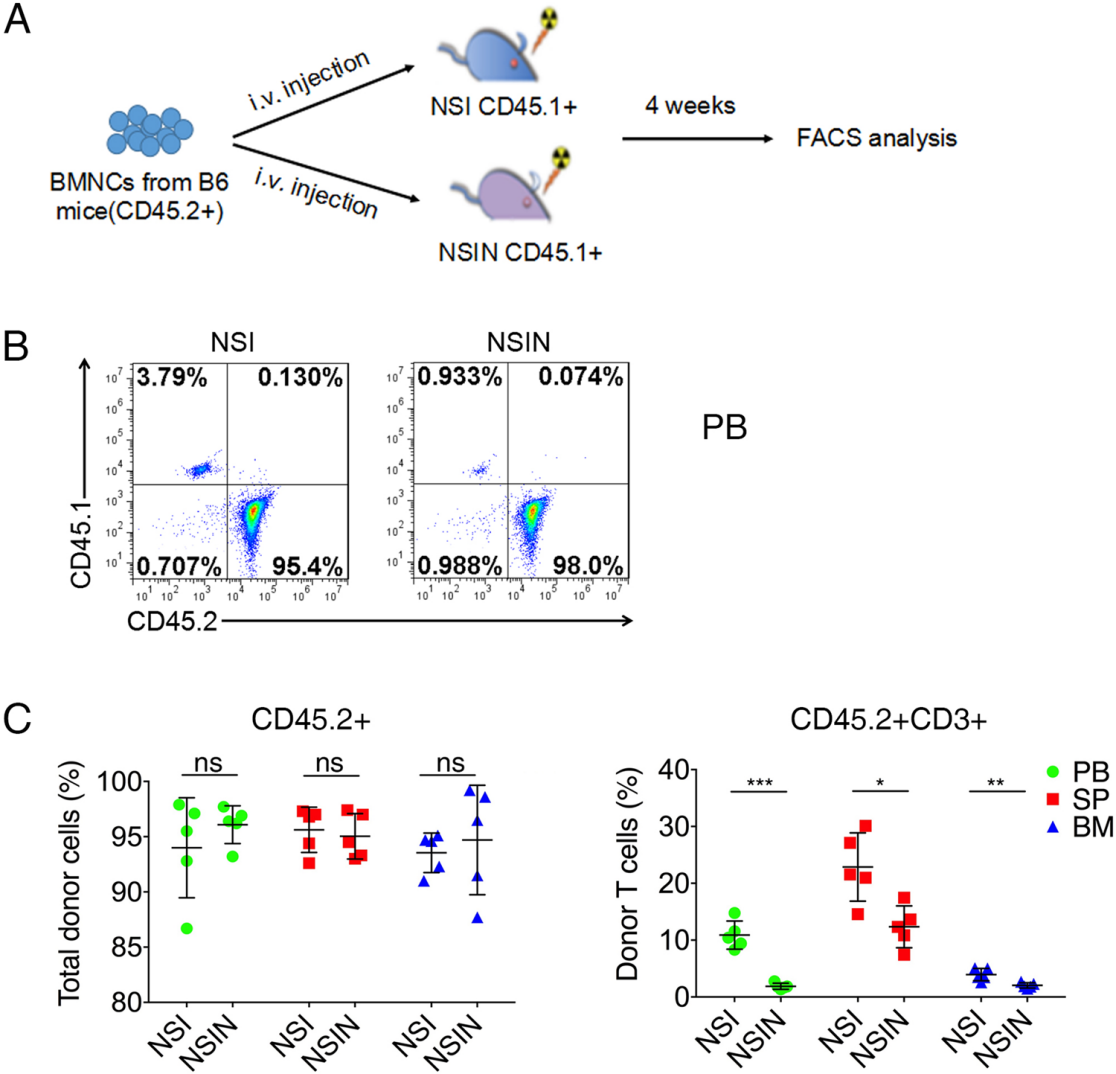
Allogeneic T cell development is affected in NSIN mice. (**A**) Schematic of the bone marrow transplantation (BMT). Bone marrow nucleated cells (BMNCs; 4 × 10^6^) from C57BL/6J (B6) donor mice (CD45.2^+^) were injected into the retro-orbital venous sinus of lethally irradiated (2 Gy) NSI (CD45.1^+^) and NSIN (CD45.1^+^) mice. The engraftment of CD45.2^+^ donor cells was measured every week in the PB of recipients. Four weeks later, five animals in each group were euthanized to analyze the repopulation efficiency of donor cells and the development of donor T cells in the recipients. (**B**) At 4 weeks, BMNCs from B6 mice reconstituted to similar levels in the PB of both NSI and NSIN mice. CD45.2^+^ cells were derived from B6 mice. (**C**) The repopulation percentages of CD45.2^+^ donor cells (left) and CD45.2^+^CD3^+^ T cells (right) in PB, SP, and BM; n = 5 for each group. Error bars denote the standard error of the mean (SEM). Groups were compared using a two-tailed unpaired *t*-test. *P < 0.05; **P < 0.01; ***P < 0.001.

*Foxn1* is a critical regulator of thymic and T cell development. To compare the capacities of adoptive T cell development between NSI and NSIN mice, we measured the reconstitution of allogeneic bone marrow nucleated cells (BMNCs) (Fig. 3A). After 4 weeks, the reconstitution of total donor cells was similar between NSI and NSIN mice (Fig. 3B); however, T cell development from BMNCs was markedly impaired in NSIN mice compared with NSI mice (Fig. 3C), as indicated by the percentages of donor T cells in the peripheral blood (PB) (Supplemental Fig. 1A), spleen (SP), and bone marrow (BM). Moreover, both CD4 and CD8 T cells were present in NSIN and NSI mice, but the CD4^−^CD8^−^ compartment was increased in NSIN mice compared with NSI mice (Supplemental Fig. 1B). Strikingly, 4 weeks after BMNC transplantation, thymi were regenerated in NSI mice, but not in NSIN mice (Supplemental Fig. 1C), confirming that *Foxn1* is a critical regulator of thymic development. In the regenerated thymus, most thymocytes were derived from the donor mice, and most T cells were CD4^+^CD8^+^ (Supplemental Fig. 1D).

### *Foxn1* deletion results in improved engraftment of leukemic cells in NSIN mice

**Figure 4:**
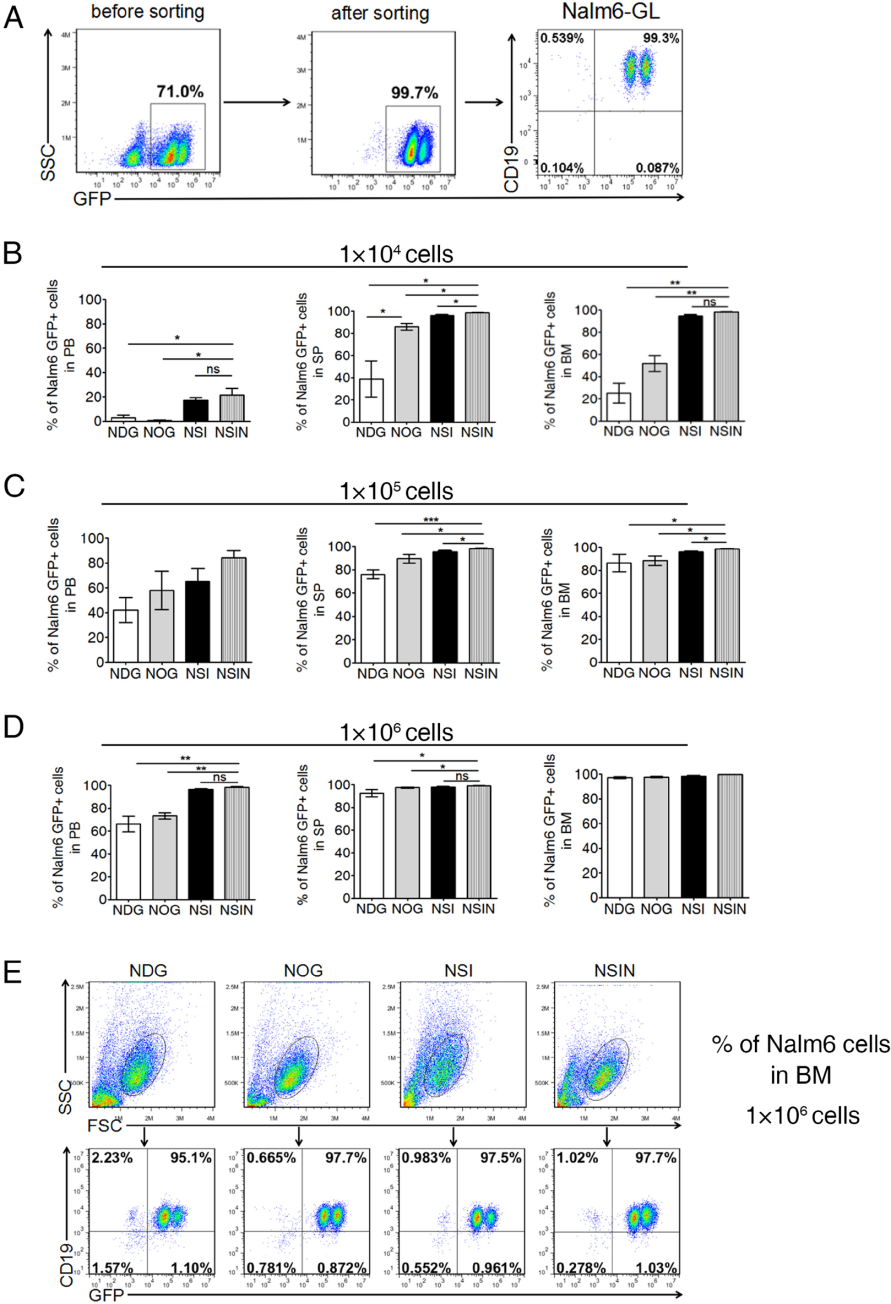
Engraftment efficiencies of Nalm6-GL cells in NDG, NOG, NSI, and NSIN mice. (**A**) Establishment of the Nalm6-GL cell line. Nalm6 cells were transfected with luciferase-GFP-expressing lentiviral vectors and sorted on GFP^+^ cells. Nalm6-GL cells were labeled for human CD19^+^ and GFP^+^. (**B**–**D**) Percentages of Nalm6-GL cells in the PB, SP, and BM of NDG, NOG, NSI, and NSIN mice injected with (**B**) low (1 × 10^4^, n = 5 for each strain), (**C**) medium (1 × 10^5^, n = 5 for each strain), and (**D**) high numbers (1 × 10^6^, n = 5 for each strain) of Nalm6-GL cells. Nalm6-GL cells were injected into each mouse through the tail vein and analyzed on day 20, 24, or 32 for the low-, medium-, and high-dose groups, respectively. (**E**) Engraftment efficiencies of Nalm6-GL cells were further confirmed by labeling with human CD19^+^ in the BM from the high-dose (1 × 10^6^) group. Cells were gated from FSC/SSC. Error bars denote the standard error of the mean (SEM). Groups were compared using a two-tailed unpaired *t*-test. *P < 0.05; **P < 0.01; ***P < 0.001.

Immunodeficiency is positively correlated with tumor engraftment capacity^12, 34, 35^. NOD/SCID/IL2rg^−/−^ mice have been widely used in translational studies of human immunology and hematological malignancies^36^. To evaluate the effect of *Foxn1* deletion on the engraftment capacity of hematological cells, we compared the engraftment of a human B cell acute lymphoblastic leukemia (B-ALL) cell line, Nalm6, in NDG, NOG, NSI, and NSIN mice. First, Nalm6 cells were labeled with green fluorescent protein (GFP)-luciferase (GL) using lentiviral transfection, followed by the FACS sorting of GFP^+^ cells (Fig. 4A). Subsequently, Nalm6-GL cells were injected into NDG, NOG, NSI, and NSIN mice at low (n = 5, for each strain) (Fig. 4B), medium (n = 5, for each strain) (Fig. 4C), and high (n = 5, for each strain) (Fig. 4D) doses via the tail vein without preconditioning. Upon exhibiting symptoms of paralysis, the mice were euthanized. The proportions of GFP^+^ Nalm6-GL cells in the PB, SP, and BM were higher in the NSIN mice than in the NDG, NOG, and NSI mice (Fig. 4B–D, Supplementary Figure 2). To ensure that GFP^+^ cells were Nalm6-GL cells, we also analyzed human CD19 and found that all GFP^+^ cells were tumor cells (Fig. 4E). Thus, the NSIN mice exhibited an enhanced capacity to engraft leukemic cells compared with the NDG, NOG, and NSI mice.

### *Foxn1* deletion in NSIN mice results in improved engraftment of solid tumors

**Figure 5:**
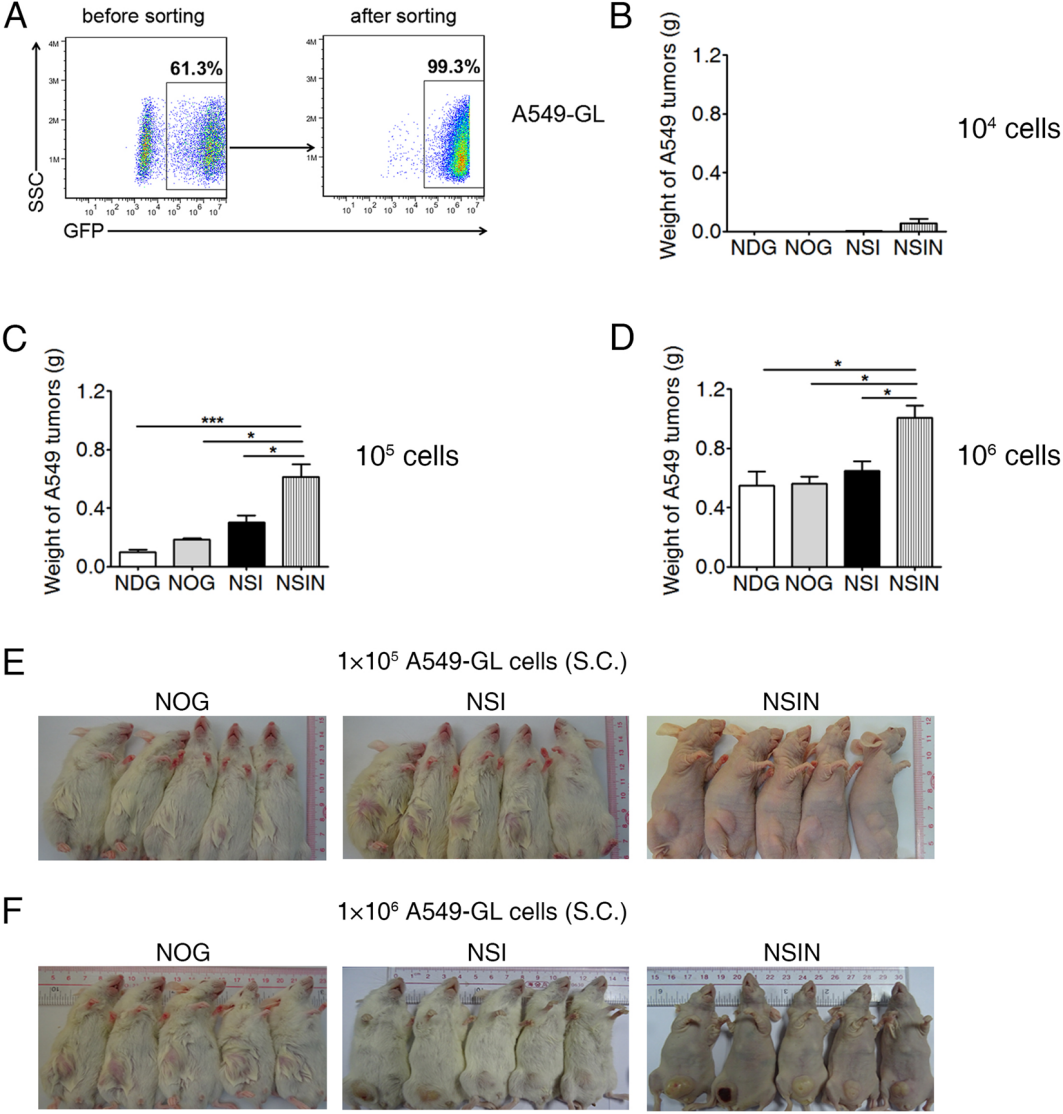
Engraftment efficiencies of A549-GL cells in NDG, NOG, NSI, and NSIN mice. (**A**) Establishment of the A549-GL cell line. Lung adenocarcinoma A549 cells were transfected with luciferase-GFP-expressing lentiviral vectors and sorted on GFP^+^ cells. (**B**–**D**) Weight of A549 tumors in NDG, NOG, NSI, and NSIN mice subcutaneously injected with (**B**) low (1 × 10^4^, n = 5 for each strain), (**C**) medium (1 × 10^5^, n = 5 for each strain), and (**D**) high numbers (1 × 10^6^, n = 5 for each strain) of A549-GL cells and analyzed on day 30. (**E**,**F**) Photographs of NOG, NSI, and NSIN mice bearing subcutaneous A549-GL tumors from the (**E**) medium- (n = 5 for each strain) and (**F**) high-dose groups (n = 5 for each strain) on day 30. The mice in the low-dose group bore no visible tumors and are not shown. Error bars denote the standard error of the mean (SEM). Groups were compared using a two-tailed unpaired *t*-test. *P < 0.05; **P < 0.01; ***P < 0.001.

To evaluate the solid tumor engraftment capacity of NSIN mice, we compared the engraftment of a human lung adenocarcinoma cell line, A549, in NDG, NOG, NSI, and NSIN mice. A549-GL cells were generated similarly to Nalm6-GL cells (Fig. 5A). A549-GL cells were subcutaneously injected into NDG, NOG, NSI, and NSIN mice at low (n = 5, for each strain), medium (n = 5, for each strain), and high doses (n = 5, for each strain). The A549-GL tumors were significantly larger in the NSIN mice than in the NDG, NOG, and NSI mice at all three doses (Fig. 5B–D). NOG, NSI, and NSIN mice bearing subcutaneous A549-GL tumors in the medium- and high-dose groups are shown (Fig. 5E,F), and tumors were most clearly visible in the NSIN mice. In contrast to the NOG and NSI mice, the blood vessels of the tumors were also clearly visible in the NSIN mice.

### NSIN mice exhibit higher tumor engraftment index (TEI) scores

**Figure 6:**
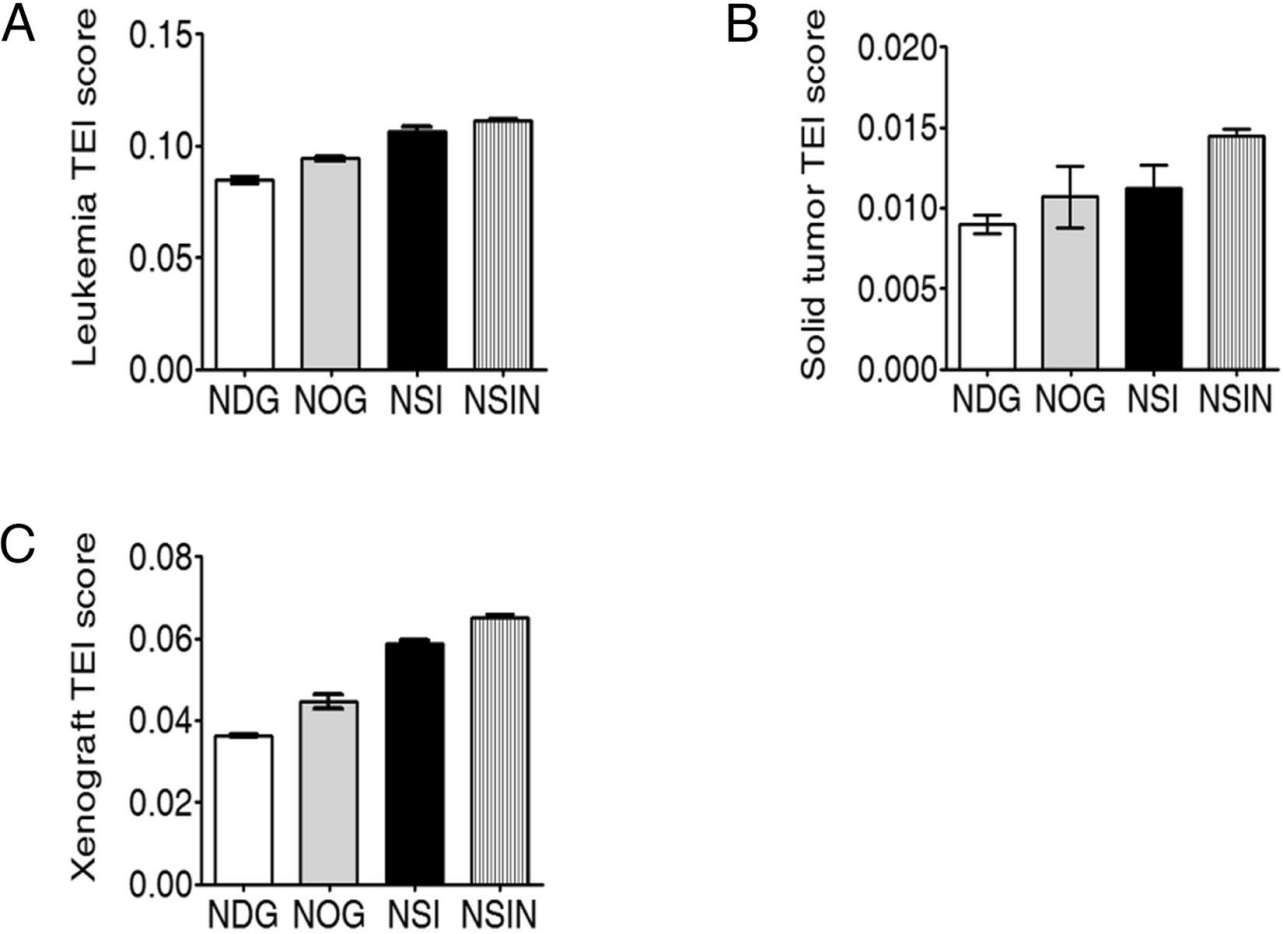
NSIN mice show higher tumor engraftment index (TEI) scores than NDG, NOG, and NSI mice. (**A**) TEI scores of NDG, NOG, NSI, and NSIN mice for Nalm6 cells. (**B**) TEI scores of NDG, NOG, NSI, and NSIN mice for A549 cells. (**C**) Overall TEI scores of NDG, NOG, NSI, and NSIN mice for both leukemia and solid tumors. Xenograft TEI scores as indicators of immunodeficiency.

Previously, we developed a TEI and a statistical formula^15^ for the simple and accurate quantification of the immunodeficiency of mouse strains. The TEI scores for NDG, NOG, NSI, and NSIN mice engrafting Nalm6-GL cells were calculated as previously described^15^ (Fig. 6A). The differences between the TEI scores of these three strains were small, indicating significant engraftment of human acute leukemia cells by these strains, consistent with previous results^12^. However, the TEI score of the NSIN mice engrafting A549-GL cells was higher than those of the NDG, NOG, and NSI mice (Fig. 6B). The overall TEI scores for hematological and solid cancers were also calculated, which indicated an overall increase in the immunodeficiency of the NSIN mice compared with the NDG, NOG, and NSI mice (Fig. 6C).

### NSIN mice facilitate live fluorescence imaging of xenografts

**Figure 7:**
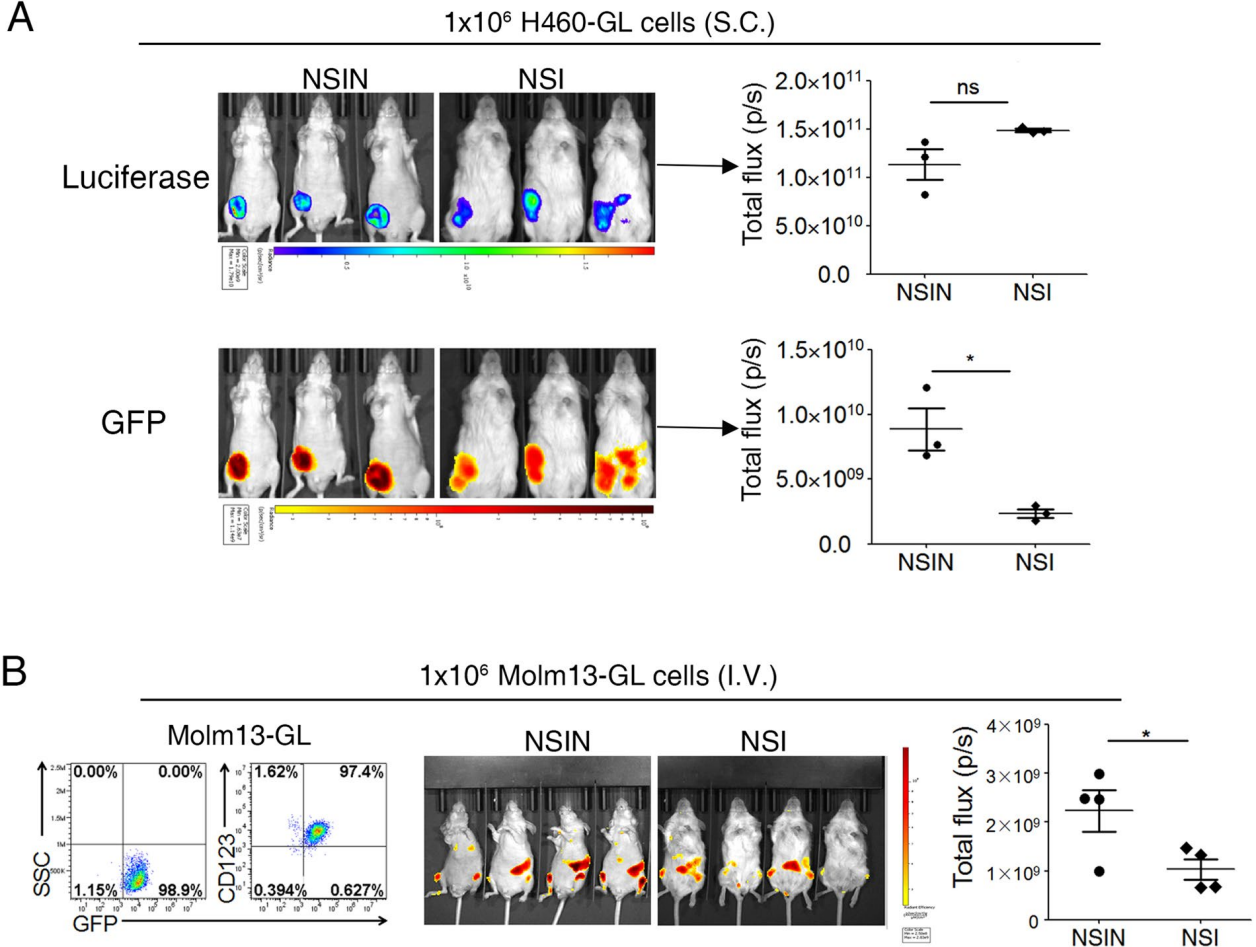
Improved live fluorescence imaging in NSIN mice. (**A**) Live fluorescence imaging of H460-GFP and luciferase (GL)-burdened mice. H460-GL cells (1 × 10^6^) were subcutaneously injected into NSIN and NSI mice (n = 3 for each group), and luciferase and GFP were analyzed on day 30. Total flux of luciferase-derived and GFP-derived fluorescence was compared between NSI and NSIN mice (right panel). H460-GL, a human large cell lung cancer cell line, expresses GL. (**B**) Live fluorescence imaging of Molm13-GL-burdened mice. Molm13-GL cells (1 × 10^6^) were injected into NSIN and NSI mice (n = 4 for each group) through the tail vein, and GFP fluorescence was analyzed on day 30. Left, the acute myeloid leukemia cell line Molm13-GL expresses CD123 and GFP; middle, live fluorescence imaging of Molm13-GL cells; right, comparison of the total flux of GFP fluorescence between NSI and NSIN mice. Error bars denote the standard error of the mean (SEM). Groups were compared using a two-tailed unpaired *t*-test. *P < 0.05; **P < 0.01; ***P < 0.001.

The hairless phenotype of NSIN mice could be advantageous in tumor monitoring and imaging. Indeed, the cells of another human large cell lung cancer cell line, H460-GL, were injected into NSIN and NSI mice, and there were no differences in the subcutaneous tumors between the NSIN and NSI mice in terms of tumor size or luciferase-derived bioluminescence. However, tumors in NSIN mice showed more condensed and sharper GFP fluorescence than those in NSI mice (Fig. 7A). Likewise, CD123^+^ human acute myeloid leukemia (AML) Molm13-GL cells were intravenously injected into NSIN and NSI mice, and GFP signals emanating from the SP and BM of the NSIN mice were more distinct than those of the NSI mice. Moreover, the tumor burden of Molm13-GL cells was higher in the NSIN mice than in the NSI mice (Fig. 7B). These results indicated that NSIN mice are more suitable hosts for the live imaging of autofluorescence derived from xenografts.

## Discussion

In basic and translational research, it is often desirable to analyze complex biological processes *in vivo*, which has led to a demand for new animal models. NSI mice have been used in translational biomedical research in our lab. However, a nude strain with comparable immunodeficiency has not been previously reported. In the present study, we generated nude NSI mice using the CRISPR/Cas9 system in the background of NSI mice via embryonic co-microinjection with Cas9 mRNA and gRNA. The mutant offspring were mated to generate homozygous NSIN mice. These mice were hairless and exhibited more severe immunodeficiency than NSI mice. Hence, NSIN mice provide a new platform for basic and translational research.

Athymic nude mice with a deletion of *Foxn1* have been widely used in cancer research. *Foxn1* is required to maintain the postnatal thymus, and changes in *Foxn1* expression in TECs may contribute to thymus involution during aging^24^. Our study also revealed that the loss of *Foxn1* resulted in the blockade of thymic development following allogeneic BMNC transplantation, which confirmed a critical role for *Foxn1* in thymic function. The postnatal thymus is the primary source of T cells in vertebrates, and thymocyte development requires interactions with TECs. Specifically, *Foxn1* controls the transcription of genes involved in the attraction and lineage commitment of T cell precursors and regulates the expression of genes involved in antigen processing and thymocyte selection^25^. Consistently, T cell development from allogeneic BMNC in NSIN mice was also impaired by the deletion of *Foxn1* in our study. Extrathymic T cell development is well established^37^ and could be responsible for the reduced but still present level of T cells in NSIN mice, especially in the SP.

Nude mice are suitable for research requiring whole-body fluorescence-based imaging techniques^38–40^. The novel NSIN strain derived using the CRISPR/Cas9 system exhibited a hairless phenotype. This was advantageous for the observation and imaging of tumors by GFP or other fluorescence. For example, the blood vessels of subcutaneous tumors could be seen through the skin of NSIN mice, making NSIN mice a potentially ideal model for xenograft angiogenesis studies. In addition, NSIN mice exhibited higher immunodeficiency than NSI mice, as indicated by their TEI scores. The elevation of TEI scores caused by *Foxn1* deletion was more significant for subcutaneous solid tumors than for hematological cancers (Fig. 5). In our study, NSI mice engrafted tumors more efficiently than NOG mice, which could be attributed to the presence of the extracellular domain of IL2rg in NOG mice but the complete deletion of IL2rg in NSI mice^3^. *Foxn1* inactivity in nude mice maintains the skin in an immature state resembling neoteny^41–43^. Thus, NSIN mice may also be useful for generating humanized skin models to study human skin regeneration.

In sum, our study generated a new mouse strain and demonstrated the advantages of this strain in xenograft model generation and tumor surveillance.

## Materials and Methods

### Mice

All animal experiments were performed in the Laboratory Animal Center of the Guangzhou Institutes of Biomedicine and Health (GIBH). All experimental protocols were performed in accordance with the instructional guidelines of the China Council on Animal Care and were approved by the Ethics Committee of Animal Experiments at GIBH. C57BL/6J (B6) (RRID: IMSR_JAX:000664), ICR/HaJ (RRID: IMSR_JAX:009122), and NOG mice were purchased from Vital River Laboratory Animal Technology Co. (Beijing, China). Another NOD/SCID/IL2rg^−/−^ strain, NDG mice, was purchased from Biocytogen (Beijing, China). NSI mice were generated by our group at GIBH^15–19^. NSIN mice were generated using CRISPR/Cas9, as detailed in this study. All mice were bred and maintained in specific pathogen-free (SPF)-grade cages and provided autoclaved food and water.

### DNA constructs

The guide sequence was incorporated into the first exon of the mouse *Foxn1* locus. A pair of oligonucleotides for the targeting site (forward: 5′-caccggaagtgtgacgtcagactg-3′; reverse: 5′-aaaccagtctgacgtcacacttcc-3′) was annealed and ligated into the BbsI site of the gRNA cloning vector (Addgene: 41824). A pcDNA3.3-hCas9 plasmid (Addgene: 41815) was used for *in vitro* transfection. A pair of oligonucleotides for the targeting site (forward: 5′-ataggaagtgtgacgtcagactg-3′; reverse: 5′-taaaaccagtctgacgtcacacttcc-3′) was annealed and ligated into the BbsI-linearized pUC57-T7 vector (Addgene: 51306) using a T7 promoter for *in vitro* transcription. The Cas9 expression vector (MLM3613; Addgene: 42251) was used for *in vitro* transcription. All plasmids were provided by Professor Liangxue Lai at GIBH.

### Cell culture and transfection

PL08 is an immortalized murine fetal liver cell line that has been previously reported^16^. Millicell Hanging Cell Culture Inserts (Millipore, Darmstadt, Germany) were used to culture PL08 cells. PL08 cells (1 × 10^6^) were transfected with U6-sgRNA vectors (10 μg) and pcDNA3.3-hCas9 plasmids (30 μg). Cells were selected using G418 (500 μg/ml; Sigma) after 24 h of transduction and were maintained in selection medium for 72 h. Single clones were collected and seeded into each well of a 96-well plate. Monoclonal cells were expanded. Then, monoclonal cell DNA was extracted to determine the cell genotypes by DNA sequencing. Nalm6, Molm13, A549, and H460 cells were cultured in RPMI-1640 media (Gibco, Grand Island, NY, USA) with 10% fetal bovine serum (FBS; Biochrom, Australia) and 1% penicillin/streptomycin. HEK-293T cells were used for lentivirus production and cultured in Dulbecco’s Modified Eagle’s Medium (Gibco) supplemented with 10% FBS and 1% penicillin/streptomycin. Lentiviral particles were produced in HEK-293T cells via polyethyleneimine (Sigma-Aldrich, St. Louis, MO, USA) transfection. A pWPXLd-based (luciferase-2A-GFP) lentiviral plasmid and two packaging plasmids, psPAX2 and pMD.2G, were co-transduced into HEK-293T cells. The supernatant containing luciferase-2A-GFP was filtered through a 0.45-μm filter and then used to transfect the Nalm6, Molm13, A549, and H460 cells. The Nalm6-GL (GFP-luciferase), Molm13-GL, A549-GL, and H460-GL cells were sorted using a FACSAria™ II (Becton Dickinson, San Jose, CA, USA) for culture.

### Transcription *in vitro*

The Cas9 expression vector (MLM3613) was linearized with Pme I (Thermo Fisher Scientific, Waltham, MA, USA) and used as the template for *in vitro* transcription (IVT) using an mMESSAGE mMACHINE T7 ULTRA Transcription Kit (Ambion, AM1345). Cas9 mRNA was purified using an RNeasy Mini Kit (Qiagen, 74104). The gRNA templates for the *in vitro* transcription of RNA were purified PCR products obtained from pUC57-T7-*Foxn1*-gRNA vectors using a primer pair (T7-F: 5′-gaaattaatacgactcactata-3′ and T7-R: 5′-aaaaccgactcggtgccacaaaagc-3′) and a high-fidelity enzyme (Takara). The T7-sgRNA PCR product was gel-purified and used as the template for IVT with a MEGAshortscript T7 Transcription Kit (Ambion, AM1354). The gRNA was purified using a MEGAclear Kit (Ambion, AM1908) and concentrated by alcohol precipitation.

### Microinjection of single-cell embryos

NSI and ICR/HaJ mouse strains were used as zygote donors and foster mothers, respectively. Female NSI mice (aged 8–10 w) were super-ovulated using intraperitoneal injections of pregnant mare serum gonadotropin (5 IU; Sigma) and human chorionic gonadotropin (5 IU; Sigma) at 48-h intervals. The super-ovulated female mice were mated to NSI male mice, and the fertilized embryos were collected from the oviducts. Cas9 mRNA (20 ng/μl) and sgRNA (20 ng/μl) were injected into the cytoplasm of fertilized eggs with visible pronuclei in M2 medium (Sigma) using a piezo-driven micromanipulator. The injected zygotes were cultured in potassium simplex optimized medium with amino acids at 37 °C under 5% CO_2_ in air until the blastocyst stage (3.5 d). The surviving embryos were selected and transferred into the uterus of pseudo-pregnant foster mothers. The resulting offspring were analyzed for edited *Foxn1* genes.

### DNA extraction and genotyping

Monoclonal cells and genomic DNA from mouse tail samples were extracted using a Universal Genomic DNA Extraction Kit Ver.3.0 (DV811A, Takara, Japan) according to the manufacturer’s instructions. Genomic DNA was subjected to PCR amplification, and mutations were identified by direct sequencing. For sequencing, the primer pair of *Foxn1*-F1 (5′-aatttctcaccttggctatc-3′)/*Foxn1*-R1 (5′-caggagtcccaaagtgacgg-3′) was used to amplify 644-bp PCR products spanning the target site. After identifying the mutation site, the primer pair *Foxn1*-F2 (5′-ttcgaggccaggactgggtg-3′)/*Foxn1*-R2 (5′-ttacgttctgtggggcaggg-3′) was used to amplify 123-bp PCR products spanning the mutation site. The PCR products were digested with the *Alu*I enzyme (Fermentas; Thermo Fisher Scientific).

### FACS analysis

Cells isolated from the PB, BM, and SP of mice were subjected to flow cytometric analyses. The cells were labeled with FACS antibodies (anti-mCD19-PE, anti-mCD3-APC, anti-mNKp46-PE, anti-mCD3-FITC, anti-mCD4-PE, anti-mCD8-Percp-cy5.5, anti-mCD45.1-PE, anti-mCD45.2-APC) on ice for 20 min. All of the antibodies were obtained from eBioscience (San Diego, CA, USA). Nalm6-GL cells were labeled with anti-hCD19-APC, and Molm13-GL cells were labeled with anti-hCD123-APC. Flow cytometric analyses were performed using a FACSAria™II. All data were analyzed using FlowJo software (Tree Star, Inc., Ashland, OR, USA).

### BMT

BMNCs (4 × 10^6^) from C57BL/6J (B6) donor mice (CD45.2^+^) were injected into the retro-orbital venous sinus of lethally irradiated (2 Gy) NSI (CD45.1^+^) and NSIN (CD45.1^+^) mice, respectively. The engraftment of CD45.2^+^ donor cells was detected weekly in the PB of recipients. Four weeks after BMT, five animals in each group were euthanized to analyze the repopulation efficiency of donor cells and the development of donor T cells in the recipients.

### Engraftment of leukemia and solid tumors

To directly compare the efficiency of cancer cell engraftment, 1 × 10^4^ (L), 1 × 10^5^ (M), or 1 × 10^6^ (H) NALM6-GL cells suspended in phosphate buffer solution (PBS; 0.2 mL) were injected into the tail veins of NOG, NSI and NSIN mice. Similarly, 1 × 10^4^(L), 1 × 10^5^(M), or 1 × 10^6^(H) A549-GL cells suspended in PBS (0.2 mL) were injected subcutaneously into NOG, NSI, and NSIN mice. The end points were based on animal models in widespread use. The engraftment of leukemia was measured by analyzing the GFP^+^ cells in the PB either weekly or when the mice were moribund after grafting.

### Live fluorescence imaging

*In vivo* whole-body imaging of GFP-labeled cells was performed using a cooled CCD camera system (IVIS 100 Series Imaging System, Xenogen, Alameda, CA, USA). Before *in vivo* imaging, mice injected with H460-GL cells or Molm13-GL cells were anesthetized with isoflurane. Quantification of total and average emissions was performed using Living Image software (Xenogen).

### Quantification of mouse immunodeficiencies (TEI)

The hematologic TEI was calculated using the following equation: *I*_strain−hematologic tumor−*n*_ = Gi/Di, where “strain” is the immunodeficient mouse strain, “hematologic tumor” is the type of tumor cells, *n* is the number of individuals, “Gi” is the sum of the percentages of tumor cells in the peripheral blood (GPB), bone marrow (GBM), and spleen (GSP) of an individual mouse either during morbidity or after death, and “Di” is the lifespan of the individual mouse after injection of the tumor cells. The solid TEI was calculated using the following equation: *I*_strain−solid tumor−*n*_ = Wi/D, where “strain” is the immunodeficient mouse strain, “solid tumor” is the type of transplanted tumor cells, *n* is the number of individuals, “Wi” is the weight of the graft in an individual mouse either during morbidity or after death, and “D” is the survival time of the mouse after injection of the tumor cells. The total TEI score was determined as follows: *I*_strain−xenograft*−n*_ = (*I*_strain−Nalm6−*n*_ + *I*_strain−A549*−n*_)/2. Calculations were performed using tools on the website of *In Vivo* Biomedical (http://www.nsitei.com)^15^.

### Statistical analysis

The data are presented as the mean ± standard deviation. Student’s *t*-test was used to determine the statistical significance of differences between samples. *P* < 0.05 was considered statistically significant. All statistical analyses were performed using Prism software, version 5.0 (GraphPad, Inc., San Diego, CA, USA).

## Electronic supplementary material

Supplementary Information
