# Supplementary material for: CRISPR/Cas9-Mediated Deletion of Foxn1 in NOD/SCID/IL2rg−/− Mice Results in Severe Immunodeficiency
Source: Sci Rep. 2017 Aug 10;7:7720. doi: 10.1038/s41598-017-08337-8 (PMC5552779; doi:10.1038/s41598-017-08337-8)

CRISPR/Cas9-Mediated Deletion of *Foxn1* in NOD/SCID/IL2rg$^{-/-}$ Mice Results in Severe Immunodeficiency

Xinru Wei[1, 2, 3], Yunxin Lai[2, 3], Baiheng Li[2, 3], Le Qin[2, 3], Youdi Xu[1, 2, 3], Simiao Lin[2, 3], Suna Wang[2, 3], Qiting Wu[2, 3], Qiubin Liang[4], Guohua Huang[5], Qiuhua Deng[5], Pentao Liu[6], Donghai Wu[2, 3], Liangxue Lai[2, 3], Yao Yao[2, 3]*, Peng Li[2, 3]*

Supplementary Figure 1. NSIN mice are more resistant to allogeneic T cell development than NSI mice. (A) Donor BMNC reconstitution in the T (CD45.2^{+} CD3^{+}) cell lineage in the PB of NSIN (upper) and NSI (lower) mice (n=5 for each group) 4 weeks after bone marrow transplantation (BMT). (B) Donor T cells were analyzed for CD4 and CD8 expression by flow cytometry. (C) Loss of *Foxn1* inhibited the regeneration of thymi after BMT in NSIN mice. (D) Phenotype of cells from the regenerated thymi in NSI mice.

Supplementary Figure 2. Representative FACS analysis of Nalm6-GL cells in NOG, NSI, and NSIN mice. (A–C) Percentages of Nalm6-GL cells in the PB, SP, and BM of mice intravenously injected with (A) low (1×10^4), (B) medium (1×10^5), and (C) high (1×10^6) numbers of Nalm6-GL cells. GFP^{+} cells represent Nalm6-GL cells.

Supplementary Figure 1

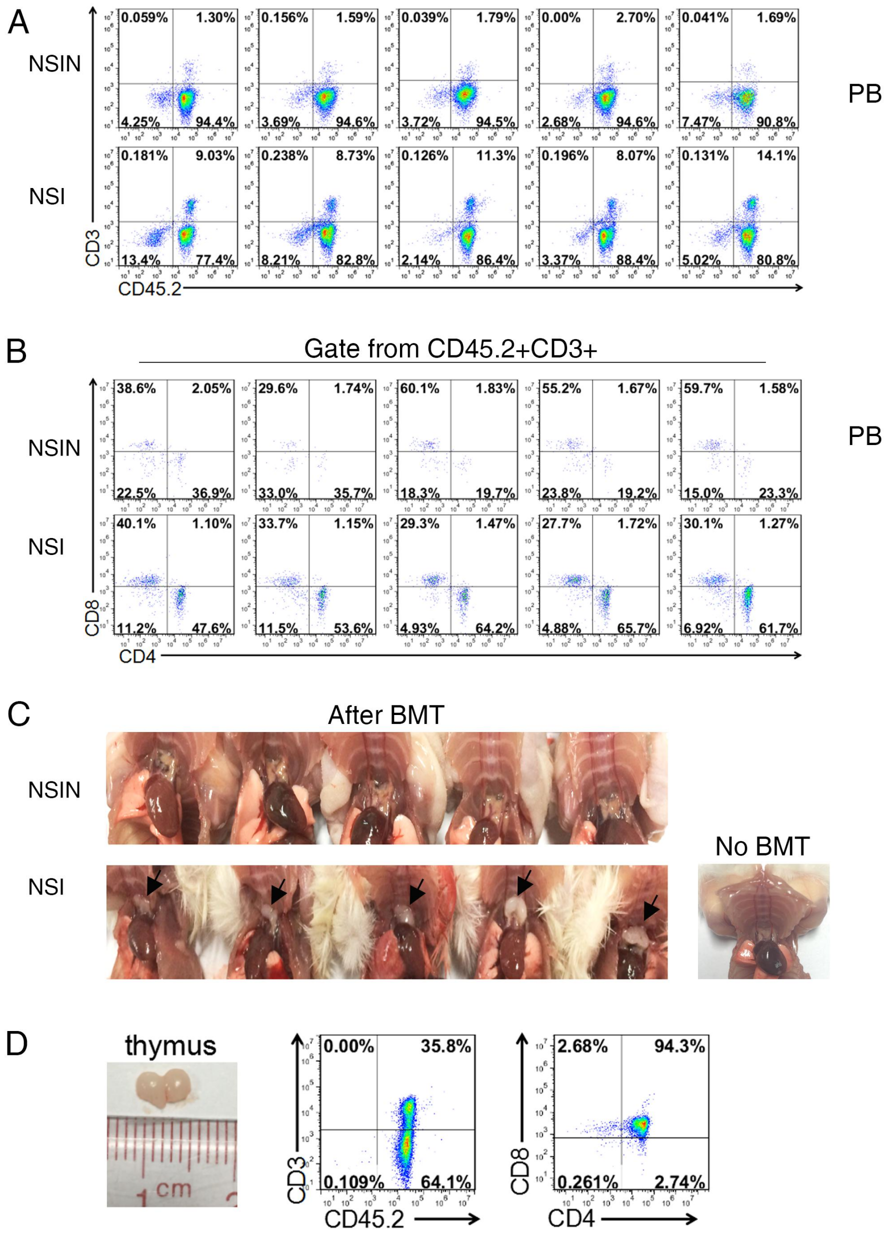

Supplementary Figure 2

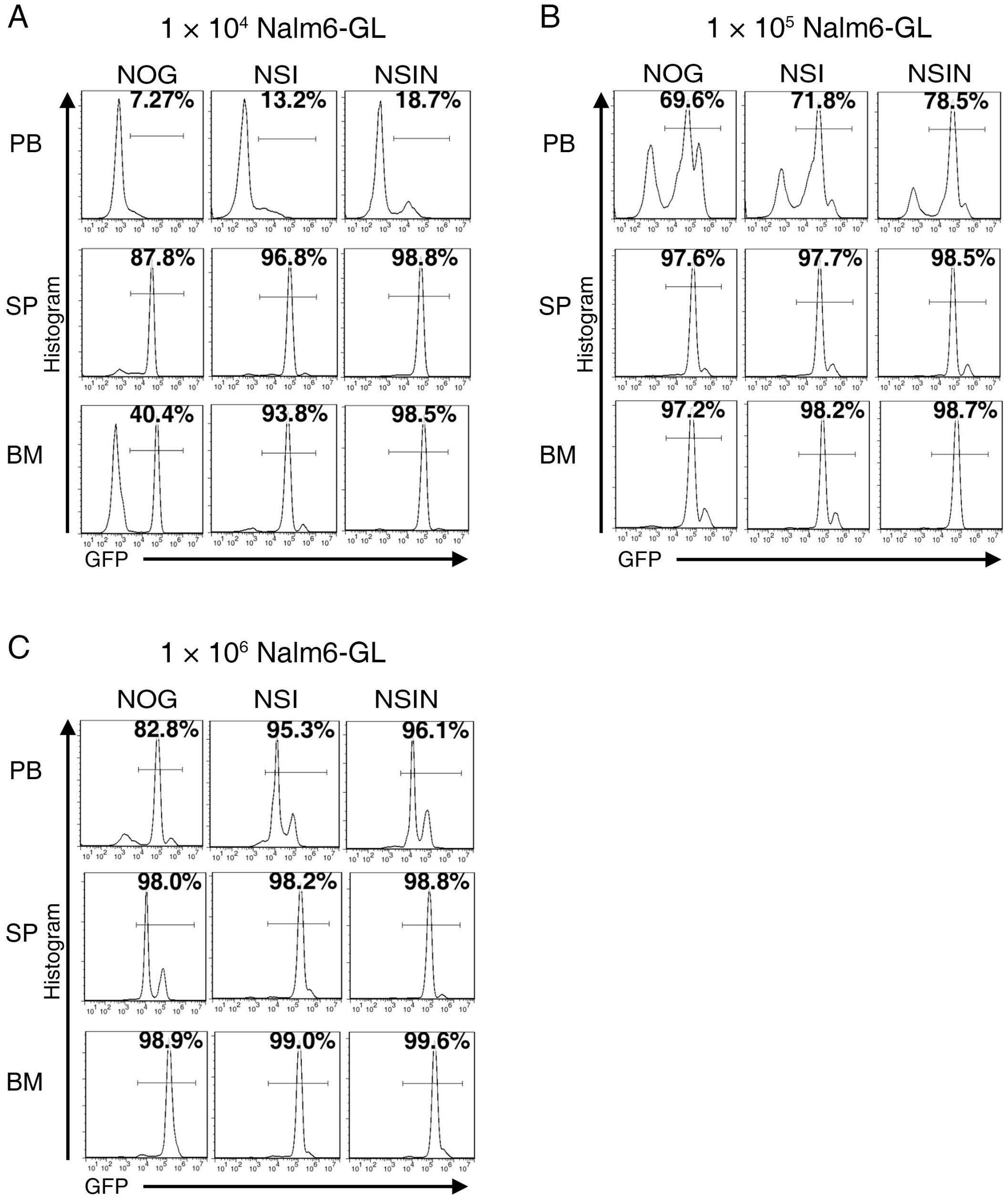

Supplement: Supplementary file 1 — Supplementary Information [file 41598_2017_8337_MOESM1_ESM.pdf]
